# Real-world experience with pertuzumab and trastuzumab combined with chemotherapy in neoadjuvant treatment for patients with early-stage HER2-positive breast cancer: the NEOPERSUR study

**DOI:** 10.1007/s12094-024-03440-5

**Published:** 2024-03-28

**Authors:** Alejandro Falcón González, Josefina Cruz Jurado, Elisenda Llabrés Valenti, Rocío Urbano Cubero, Maria Carmen Álamo de la Gala, María Antonia Martínez Guisado, Rocío Álvarez Ambite, Carlos José Rodríguez González, Marta Amérigo Góngora, Lourdes Rodríguez Pérez, Pilar López Álvarez, Pedro Sánchez Rovira, Encarnación González Flores, Fernando Henao Carrasco, Juan Bayo Calero, María Valero Arbizu, Alicia Quílez Cutillas, Javier Salvador Boffil, Eloísa Rubio Pérez, Manuel Ruiz-Borrego

**Affiliations:** 1 Medical Oncology Department, Hospital Universitario Virgen del Rocío, 41013 Seville, Spain; 2 Medical Oncology Department, Hospital Universitario de Canarias, 38320 Santa Cruz, Tenerife Spain; 3 Oncology Service, Complejo Hospitalario Universitario Insular Materno-Infantil, 35016 Las Palmas, Gran Canaria Spain; 4 Medical Oncology Department, Hospital Universitario de Jaén, 23009 Jaén, Spain; 5 Medical Oncology Department, Hospital Universitario Virgen Macarena, 41009 Seville, Spain; 6 Oncology Department, Complejo Hospitalario Torrecárdenas de Almería, 04009 Almería, Spain; 7 Medical Oncology Department, Hospital Universitario San Cecilio, 18016 Granada, Spain; 8 Medical Oncology Department, Hospital Juan Ramón Jiménez, 21005 Huelva, Spain; 9 Medical Oncology Department, Hospital Universitario Puerta del Mar, 11009 Cádiz, Spain; 10 Medical Oncology Department, Hospital Universitario Virgen de Valme, 41014 Seville, Spain; 11 Medical Oncology Unit, Hospital Universitario Virgen de las Nieves, 18014 Granada, Spain; 12 Medical Oncology Unit, Oncoavanze-Hospitales Quirónsalud, 41013 Seville, Spain; 13 Medical Oncology Department, Hospital Universitario Puerto Real, 11510 Puerto Real, Cádiz Spain; 14 Methodological and Statistical Management Unit, Fundación Pública Andaluza Para La Gestión de La Investigación en Salud de Sevilla (FISEVI), Hospital Universitario Virgen del Rocío, 41013 Seville, Spain

**Keywords:** Pertuzumab, Trastuzumab, Breast cancer, Early stage, HER2, Real-world data

## Abstract

**Purpose:**

HER2-targeted therapies have dramatically improved outcomes of patients with HER2-positive breast cancer (BC), as demonstrated in neoadjuvant trials. This study aims to provide real-world evidence on the use and effectiveness of combined pertuzumab, trastuzumab and chemotherapy (CT) in early-stage HER2-positive BC.

**Methods:**

A retrospective, multicentre study was conducted on patients diagnosed with HER2-positive early BC treated with neoadjuvant pertuzumab and trastuzumab plus CT at 13 Spanish sites. The primary endpoint was pathological complete response (pCR).

**Results:**

A total of 310 patients were included. Pertuzumab and trastuzumab were combined with anthracyclines and taxanes, carboplatin and docetaxel, and taxane-based CT in 77.1%, 16.5%, and 6.5% of patients, respectively. Overall, the pCR rate was 62.2%. The pCR was higher amongst patients with hormone receptor-negative tumours and with tumours expressing higher levels of Ki-67 (> 20%). After postoperative adjuvant treatment, 13.9% of patients relapsed. Those patients who did not achieve pCR, with tumours at advanced stages (III), and with node-positive disease were more likely to experience distant relapse. Median overall survival (OS) and distant disease-free survival (D-DFS) were not reached at the study end. The estimated mean OS and D-DFS times were 7.5 (95% CI 7.3–7.7) and 7.3 (95% CI 7.1–7.5) years, respectively (both were significantly longer amongst patients who achieved pCR). Grade 3–4 anti-HER2 related toxicities were reported in six (1.9%) patients.

**Conclusion:**

Neoadjuvant pertuzumab and trastuzumab plus CT achieve high pCR rates in real-life patients with HER2-positive early BC, showing an acceptable safety profile. Innovative adjuvant strategies are essential in patients at high risk of distant disease recurrence.

## Introduction

Breast cancer (BC) is the most frequently diagnosed cancer in the world, affecting more than 2 million people and causing nearly 680,000 deaths each year [1]. Amplification of HER2/*neu* gene or otherwise overexpression of the human epidermal growth factor receptor 2 (HER2) is found in approximately 15–20% of breast cancers [2] and has been classically associated with an aggressive clinical course and poor outcomes [3]. However, the development of HER2-targeted therapies in recent years has substantially improved outcomes in these patients.

The benefits of neoadjuvant BC treatment with chemotherapy (CT), endocrine therapy and/or targeted therapy are well established, as it improves tumour resectability, often reducing the extent of breast and axillary surgery [4]. In addition, neoadjuvant systemic therapy allows an approach to personalised adjuvant treatment based on the pathological response. Pathological complete response (pCR) is defined as the absence of residual invasive cancer of the complete resected breast tumour and all regional lymph nodes (ypT0/Tis ypN0), after completion of neoadjuvant systemic therapy [5]. Achievement of pCR at surgery is correlated with favourable outcomes and is considered a reliable surrogate endpoint for enhanced survival in HER2-positive BC [6].

Trastuzumab represents the cornerstone for neoadjuvant treatment in HER2-positive BC due to its success in several clinical trials. The addition of trastuzumab to conventional neoadjuvant CT showed remarkable improvements in pCR and event-free survival [7, 8]. Nevertheless, many patients are likely to develop resistance to trastuzumab [9]. Pertuzumab has a complementary mechanism of action to that of trastuzumab, which consists of binding the extracellular domain II of HER2, preventing HER2-HER3 dimerization. The NeoSphere trial showed increased pCR rates in patients treated with pertuzumab plus trastuzumab combined with CT compared to those who received only trastuzumab and CT in the neoadjuvant setting [10]. The combination of pertuzumab with trastuzumab and different CT regimens has been investigated in neoadjuvant trials, demonstrating not only enhanced pCR rates over the 60%, but also overall survival benefits [11–14].

Following these impressive outcomes, the neoadjuvant combination of CT with dual anti-HER2 therapy became the standard of care. However, there remains a significant risk of relapse for these patients.

Despite the extensive variety of clinical trials supporting the efficacy and safety of the neoadjuvant use of pertuzumab, trastuzumab plus CT in HER2-positive BC, these studies often include highly selected patient populations which could not necessarily represent the general population with early-stage HER2-positive BC. Real-world data (RWD) complement clinical trial knowledge by gathering routine clinical practise which encompasses a broader spectrum of patients. Available real-world evidence on the use of this treatment combination under routine clinical practise is still limited [15–17], highlighting the need to conduct RWD studies. The NEOPERSUR study aims to confirm whether the improvements in pCR rates after neoadjuvant treatment with dual anti-HER2 therapy for patients with early-stage HER2-positive BC observed in clinical trials are translated into a real-world setting. We also investigated patient and tumour characteristics associated with pCR achievement, pCR prognosis value, and risk factors for BC distant recurrence. Our study will provide further knowledge on pCR after neoadjuvant treatment, and real-life patient characteristics associated with pCR achievement and risk for BC distant relapse. Altogether, this will bring on a step forward to improve neoadjuvant and adjuvant treatment decision-making for these patients.

## Methods

### Study design and patients

The objectives of the NEOPERSUR study were to investigate the effectiveness of the neoadjuvant dual HER2-blockade with pertuzumab and trastuzumab combined with CT in the achievement of pCR in real-life patients with early-stage HER2-positive BC, and to describe patient characteristics associated with pCR achievement and risk for distant relapse. To accomplish these objectives, we conducted a retrospective medical chart review of patients with early-stage HER2-positive BC who had been treated with neoadjuvant pertuzumab, trastuzumab and CT and subsequent surgery at 13 Spanish hospitals.

Adult women (≥ 18 years) with histologically confirmed HER2-positive localised or locoregionally advanced breast cancer (i.e. 3+ result by immunohistochemistry [IHC], or 2+ result by IHC and positive result by fluorescence in situ hybridization [FISH]), who received neoadjuvant combination treatment with pertuzumab, trastuzumab and chemotherapy (CT), and who had undergone surgery up to December 2018 (with an available anatomopathological report), were included in the study. Patients were excluded if they had received this neoadjuvant combination in the context of a clinical trial or as off-label treatment. Data from the Oncology departments were retrospectively collected from BC diagnosis until December 2022.

This study was approved by the Ethics Committee of the Hospital Universitario Virgen Macarena (Seville, Spain). The study was conducted in accordance with the ethical principles of the Declaration of Helsinki, Good Clinical Practice (GCP), and in compliance with European and local requirements. Written informed consent was not required in accordance with the national legislation (Real Decreto 957/2020).

### Study endpoints

The primary endpoint was the pCR rate in the breast and axillary lymph nodes (ypT0/Tis ypN0) by local pathology assessment. The pathological response was evaluated using the Miller–Payne grading system [18] as per routine clinical practise. The definition of pCR was the absence of tumour cells of the complete resected breast tumour and axillary lymph nodes, after completion of neoadjuvant systemic therapy. Secondary endpoints included the demographic and clinical characteristics of patients, description of neoadjuvant and adjuvant treatment schemes, distant recurrence rate, distant disease-free survival (D-DFS), overall survival (OS), and toxicity. The OS was estimated as the time elapsed from BC diagnosis to death due to any cause or until database cut-off. The D-DFS was estimated as the free of distant disease interval from breast tumour resection (surgery) until the database cut-off; local relapses were not considered for the analyses.

### Statistical analysis

As this was an exploratory study, with a descriptive aim of collecting, summarising and providing data on the treatment of real-life patients with early-stage HER2-positive BC with neoadjuvant pertuzumab, trastuzumab and CT, and the outcomes of this treatment, no pre-specified hypothesis was made, and therefore sample size was not estimated.

A descriptive statistical analysis was performed on the study variables including calculation of measures of central tendency and dispersion (mean and standard deviation [SD], median and interquartile range [IQR]) for quantitative variables, and frequencies and valid percentages for qualitative variables. Data collected from medical records included patients’ age and menopausal status, tumour stage, tumour grade (AJCC cancer staging manual: breast cancer, 8th edition), lymph node involvement, hormone receptor (HR) status (HR-negative or HR-positive) and Ki-67 levels using the 20% cut-off (≤ 20% vs. > 20%) [19]. Comparison between groups with categorical variables was made using the Pearson’s Chi-squared test. Time-to-event endpoint analyses were estimated using the Kaplan–Meier method and compared with the log-rank test.

A logistic regression model was performed to study the association between pCR achievement/relapse and each of the possible clinical factors of interest starting with all variables that were significant in the bivariate analyses (*p* < 0.200).

Missing data were not considered in the analyses. All hypothesis tests were bilateral; significance was considered at *p* < 0.05. All statistical analyses were conducted using the Statistical Package for the Social Sciences (SPSS) version 21.0 (SPSS Inc, Chicago, IL, USA).

## Results

### Patient characteristics

A total of 310 patients diagnosed with early-stage HER2-positive BC, and treated with pertuzumab, trastuzumab plus CT met the selection criteria and were enrolled in the study. The demographic and clinical characteristics of the entire cohort are described in Table 1. Briefly, the mean (SD) age was 51.0 (11.1) years, and 54.5% were premenopausal. Grade 2 and 3 tumours were found in 143 (46.1%) and 90 (29.0%) patients, respectively, and 65.7% of patients had lymph node involvement. Overall, 51% of patients had HR-positive tumours, and 49% of patients had HR-negative disease.

**Table 1:** Baseline characteristics of patients

Characteristics	Value
Age (years), mean (SD) [*N*]	51.0 (11.1) [309]
Menopausal status, *n* (%) [*N*]	
Premenopausal	169 (54.5) [310]
Postmenopausal	141 (45.5) [310]
Tumour size (grouped), *n* (%) [*N*]	
T1	34 (11.0) [309]
T2	168 (54.4) [309]
T3	72 (23.3) [309]
T4	35 (11.3) [309]
Tumour grade, *n* (%) [*N*]	
G1	9 (2.9) [310]
G2	143 (46.1) [310]
G3	90 (29.0) [310]
Unknown	68 (21.9) [310]
Tumour stage, *n* (%) [*N*]	
IA	2 (0.6) [309]
IB	2 (0.6) [309]
IIA	91 (29.4) [309]
IIB	113 (36.6) [309]
IIIA	58 (18.8) [309]
IIIB	36 (11.7 [309]
IIIC	7 (2.3) [309]
Lymph node involvement^a^, *n* (%) [*N*]	
N0	106 (34.3) [309]
N-positive	203 (65.7) [309]
N1	169 (83.3) [203]
N2	27 (13.3) [203]
N3	7 (3.4) [203]
Hormone receptor status, *n* (%) [*N*]	
HR-positive	158 (51.0) [310]
HR-negative	152 (49.0) [310]
Ki-67 levels, *n* (%) [*N*]	
≤ 20%	61 (20.3) [300]
> 20%	239 (79.7) [300]

*HR* hormone receptor, *IQR* interquartile range, *n* patients with event, *N* number of patients with available data, *SD* standard deviation

^a^TNM staging system

### Treatment patterns

In the neoadjuvant setting, pertuzumab and trastuzumab were mostly combined with anthracyclines and taxanes (77.1%), whilst 16.5% of patients received them combined with carboplatin and docetaxel. Only 6.5% of patients received taxane-based chemotherapy, pertuzumab and trastuzumab. Neoadjuvant treatment approaches are shown in Table 2.

**Table 2:** Treatment approaches

Treatment schemes	*n* (%) [*N*]
Neoadjuvant treatment	
Anthracyclines and taxanes + P + T	239 (77.1) [310]
Carboplatin + docetaxel + P + T	51 (16.5) [310]
Taxane-based CT + P + T	20 (6.5) [310]
Adjuvant treatment^a^	
Trastuzumab	306 (99.0) [309]
Pertuzumab	20 (6.5) [309]
Neratinib	5 (1.6) [309]
T-DM1	1 (0.3) [309]

*CT* chemotherapy, *n* patients with event, *N* number of patients with available data, *P* pertuzumab, *T* trastuzumab

^a^Multiple response. Percentages can add up to more than 100%

The majority (*n* = 309; 99.7%) of patients had available data on postoperative adjuvant therapy. Trastuzumab was the most common (99.0%), mainly (97.7%) for a period of 12 months, followed by pertuzumab (6.5%), neratinib (1.6%) and the antibody–drug conjugate ado-trastuzumab emtansine (T-DM1; 0.3%) (Table 2).

### Effectiveness

Pathological response was evaluable in almost all (*n* = 307; 99.0%) patients of the study, of whom 62.2% achieved pCR (ypT0/Tis ypN0). According to the neoadjuvant treatments received, pCR was achieved by 66.7% of patients treated with carboplatin and docetaxel combination CT, 62.0% treated with anthracyclines and taxanes, and 52.6% of patients receiving taxane-based CT (Table 3). No significant differences were found between neoadjuvant treatments in terms of pCR achievement (*p* = 0.555).

**Table 3:** Achievement of pCR in the overall population, and factors associated with pCR achievement

Bivariate analysis	pCR, *N* (%)	Residual disease, *N* (%)	*p* value^a^
Overall population (*N* = 307)	191 (62.2)	116 (37.8)	
Neoadjuvant treatment regimen			
Taxane-based CT + P + T	10 (52.6)	9 (47.4)	0.555
Anthracyclines and taxanes + P + T	147 (62.0)	90 (38.0)	
Carboplatin + docetaxel + P + T	34 (66.7)	17 (33.3)	
Hormone receptor status			
HR-positive	85 (53.8)	73 (46.2)	0.002
HR-negative	106 (71.1)	43 (28.9)	
Tumour stage			
I–II	128 (61.5)	80 (38.5)	0.771
III	62 (63.3)	36 (36.7)	
Lymph node involvement			
N0	63 (59.4)	43 (40.6)	0.485
N-positive	127 (63.5)	73 (36.5)	
Ki-67 levels			
≤ 20%	30 (49.2)	31 (50.8)	0.021
> 20%	154 (62.3)	82 (34.7)	

Multivariate analysis	OR	95% CI
Hormone receptor status	2.143	1.321–3.477
Ki-67 levels	1.931	1.089–3.445

*CI* confidence interval, *CT* chemotherapy, *HR* hormone receptor, *n* patients with event, *N* number of patients data, *OR* odds ratio, *P* pertuzumab, *pCR* pathological complete response, *T* trastuzumab

^a^Pearson’s Chi-squared test

Subgroup analyses of response are shown in Table 3. The pCR rate was significantly higher amongst patients with HR-negative disease compared to those with HR-positive tumours (71.1% vs. 53.8%; *p* = 0.002). In addition, the pCR rate was significantly superior in patients with tumours expressing higher levels of Ki-67 compared to patients with tumours expressing Ki-67 ≤ 20% (62.3% vs. 49.2%; *p* = 0.021). Achievement of pCR was not significantly associated with the tumour stage nor lymph node involvement.

Multivariate analyses revealed that a HR-negative status and higher levels of Ki-67 (> 20%) were independent predictive factors for achieving pCR in patients with early-stage HER2-positive breast cancer after being treated with the neoadjuvant combination of pertuzumab, trastuzumab and CT (Odds ratio [OR] = 2.143; 95% confidence interval [CI] 1.321–3.477, and OR = 1.931; 95% CI 1.082–3.445, respectively) (Table 3).

At the database cut-off, 43 (13.9%) patients suffered distant relapse, whether central nervous system recurrence (39.5%) or relapsing in other visceral organs or bones (60.5%). Distant recurrence was observed more frequently in those who did not achieve pCR after neoadjuvant therapy (*p* = 0.001). As displayed in Table 4, 62.8% of patients who did not achieve pCR experienced disease relapse, whilst only 37.2% of patients achieving pCR relapsed. Experiencing distant relapse was not significantly associated with neoadjuvant treatment regimens and neither with receiving adjuvant treatments neratinib and pertuzumab.

**Table 4:** Distant relapse of the disease in the overall population, and factors associated with its occurrence

Bivariate analysis	Relapse, *N* (%)	No relapse, *N* (%)	*p* value^a^
Overall population (*N* = 310)	43 (13.9)	267 (86.1)	
Neoadjuvant treatment regimen			
Taxane-based CT + P + T	5 (11.6)	15 (5.6)	0.161
Anthracyclines and taxanes + P + T	34 (79.1)	205 (76.8)	
Carboplatin + docetaxel + P + T	4 (9.3)	47 (17.6)	
Achievement of pCR			
Achieved	16 (37.2)	175 (66.2)	< 0.001
Not achieved	27 (62.8)	89 (33.7)	
Adjuvant treatment			
Neratinib			
Yes	0 (0.0)	5 (1.9)	0.365
No	43 (100.0)	261 (98.1)	
Pertuzumab			
Yes	2 (4.7)	18 (6.8)	0.601
No	41 (95.3)	248 (93.2)	
Hormone receptor status			
HR-positive	21 (48.8)	137 (51.3)	0.763
HR- negative	22 (51.2)	130 (48.7)	
Tumour stage			
I–II	17 (40.5)	191 (71.5)	< 0.001
III	25 (59.5)	76 (28.5)	
Lymph node involvement			
N0	6 (14.3)	100 (37.5)	0.003
N-positive	36 (85.7)	167 (62.5)	
Ki-67 levels			
≤ 20%	12 (29.3)	49 (18.9)	0.126
> 20%	29 (70.7)	210 (81.1)	

Multivariate analysis	OR	95% CI
Achievement of pCR	4.306	2.050–8.630
HR status	3.272	1.570–6.823
Lymph node involvement	2.730	1.036–7.193

*CI* confidence interval, *CT* chemotherapy, *HR* hormone receptor, *n* patients with event, *N* number of patients with available data, *OR* odds ratio, *P* pertuzumab, *pCR* pathological complete response, *T* trastuzumab

^a^Pearson’s Chi-squared test

The distant relapse rate was significantly higher amongst patients with tumours at more advanced stages (tumour stage I–II: 40.5%; III: 59.5%; *p* < 0.001). Similarly, patients with lymph node involvement also showed a higher relapse rate than those without affected lymph nodes (85.7% vs. 14.3%; *p* = 0.003). Distant disease recurrence was not significantly associated with Ki-67 levels or the hormone receptor status (Table 4).

Multivariate analyses exposed the lack of pCR achievement, having tumours in advanced stages (III), and the lymph node affectation as independent predictive factors for distant relapse (OR = 4.206; 95% CI 2.050–8.630, OR = 3.272; 95% CI 1.570–6.823, and OR = 2.730; 95% CI 1.036–7.193, respectively) (Table 4).

**Fig. 1:**
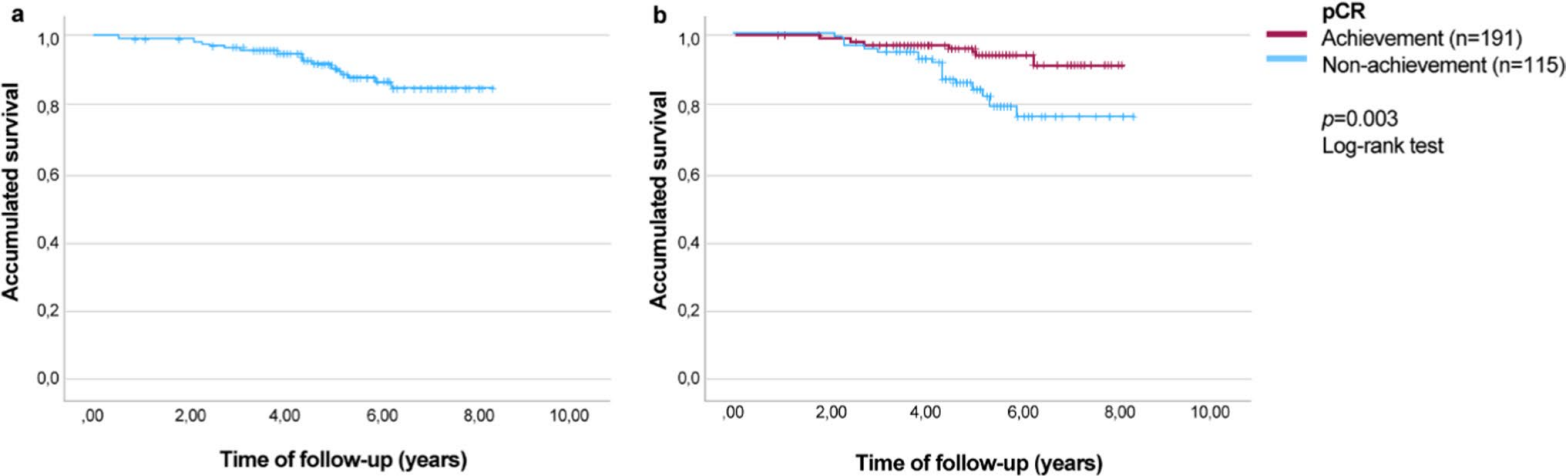
Overall survival of **a** the study population (*n* = 309), and **b** according to pathological response (pCR achievement vs. pCR non-achievement)

Median overall survival (OS) could not be calculated because more than half of patients were still living. The estimated mean OS time for the entire cohort (*n* = 309) was 7.5 years (95% confidence interval [CI] 7.3–7.7) (Fig. 1a). According to pathological response, OS was significantly longer in those patients who achieved pCR (7.6 years [95% CI 7.4–7.8] vs. 7.2 years [95% CI: 6.8–7.6]; *p* = 0.003) (Fig. 1b). Estimated survival at the end of the follow-up was 90.9% (95% CI 84.2–98.1) in patients who achieved pCR versus 75.8% (95% CI 65.3–88) in those who did not.

**Fig. 2:**
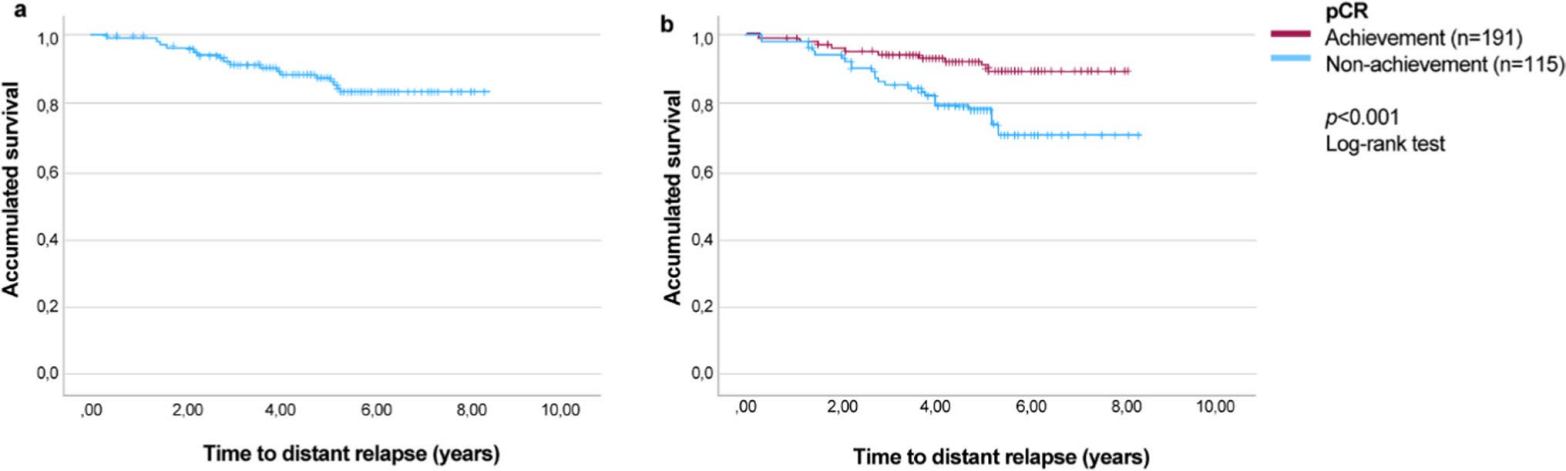
Distant disease-free survival (D-DFS) of **a** the study population (*n* = 309), and **b** according to pathological response (pCR achievement vs. pCR non-achievement)

Similar to OS, median of D-DFS was not reached at the end of the follow-up. Mean D-DFS time for the entire cohort (*n* = 309) was 7.3 (95% CI 7.1–7.5) years (Fig. 2a). According to pCR achievement, D-DFS was also significantly longer in those patients who achieved pCR (7.4 [95% CI 7.1–7.6] vs. 6.7 [95% CI 6.2–7.2]; *p* < 0.001) (Fig. 2b). Estimated D-DFS at the end of the follow-up was 89.4% (95% CI 60.3–82.5) in patients who achieved pCR versus 70.6% (95% CI 60.3–82.5) in those who did not.

### Toxicity

Overall, 264 (72.9%) patients experienced any adverse event related to chemotherapy, with 14.8% having grade 3–4 toxicities.

Most (*n* = 273, 88.1%) patients did not experience any toxicity related to anti-HER2 treatment. Grade 3–4 toxicities were reported only in six (1.9%) patients [left ventricular systolic dysfunction (LVSD) in all cases]. None of the patients who experienced cardiac toxicity restarted anti-HER2 treatment.

## Discussion

The main goal in the treatment of BC at early stages is to eliminate the tumour and prevent its recurrence. The present study shows that neoadjuvant pertuzumab and trastuzumab combined with CT achieves high pCR rates in real-life patients with early-stage HER2-positive BC. The achievement of pCR was determined by the HR status and Ki-67 levels. Our findings also support the predictive value of pCR in BC distant recurrence after neoadjuvant treatment and postoperative adjuvant therapy. A limited proportion (13.9%) of patients suffered distant relapse. We identified non-pCR achievement, having tumours in advanced stages (III), and the lymph node affectation, as risk factors for BC distant relapse.

In this real-world analysis, we observed a pCR rate of 62.2%, which is similar or even higher than pCR rates reported in previous clinical trials. In the NeoSphere trial, a pCR rate of 45% was observed in patients who received pertuzumab plus trastuzumab and docetaxel [10]. Similar to our analysis, the BERENICE [13] and the GeparSepto [11] trials showed pCR rates > 60% (61.8% and 66.2%, respectively) with neoadjuvant trastuzumab, pertuzumab and anthracycline/taxane-based CT regimens. In addition, the TRAIN-2 trial reported high pCR rates after neoadjuvant CT with or without anthracyclines (67% vs. 68%) plus dual-HER2 blockade [14]. The comparison by CT approaches is limited in clinical trials. Of note, we did not observe differences in pCR achievement according to neoadjuvant treatment patterns.

Although the pCR benefit demonstrated in the clinical trial setting has supported the neoadjuvant use of pertuzumab and trastuzumab with CT for these patients, available data of real-world patients are currently limited. An analysis of the German PRAEGNANT network and other multicentre studies reported slightly reduced pCR rates (52.8% and 46.8%, respectively) [17, 20]. More recently, in a real-life study at two Chinese institutions, Ma et al. observed an overall pCR rate of 64.9% [16], which is in line with our results. Our analysis is also consistent with the Spanish real-world study NEOPETRA (pCR rate of 66%), conducted in a cohort of 250 patients treated with neoadjuvant dual anti-HER2 therapy for early-stage HER2-positive BC [15].

The pCR rate was higher in patients with HR-negative tumours, as exposed in previous neoadjuvant studies [10, 13, 15]. We also found higher levels of Ki-67 (> 20%) as an independent predictive factor for pCR achievement. By contrast, Zhou M. and collaborators associated Ki-67 levels < 15% with pCR in these patients [17]. Although most studies have indicated that a high percentage of Ki-67 correlates to a better response in reducing the size or eliminating the breast tumours, it is still not considered as a validated marker to be used in clinical practise [21], likely because of the retrospective design of these studies and their limited sample size.

Despite of trastuzumab and other anti-HER2 drugs have led to substantial improvements in the outcomes for patients with HER2-positive early-stage BC, there remains a significant risk of recurrence. It is estimated that up to 25% of patients with early-stage HER2-positive BC treated with HER2-targeted therapy will eventually suffer a relapse within 10 years [22]. In our assessment, 13.9% of patients relapsed. Systematic literature reviews include non-achievement of pCR, residual cancer burden, and fewer tumour-infiltrating lymphocytes (TILs), as risk factors specifically identified for early-stage HER2-positive BC recurrence [23]. Differing from them, we found that patients with tumours in advanced stages (III) and the lymph node affectation were risk factors for BC distant relapse in this population. However, our results highlight the importance of the pCR status on the recurrence of the disease. Indeed, other analyses have exposed the pCR as the risk factor most closely associated with decreased risk of HER2-positive early-stage BC recurrence and long-term outcomes [6, 24]. In this regard, predictive tools as the genomic test HER2DX, which efficiently predicts pCR in early-stage HER2-positive BC [25], would be helpful to identify ideal candidates to receive neoadjuvant dual HER2-blockade plus CT to eliminate the tumour and prevent distant recurrence, and in escalating/de-escalating treatments.

Beyond the pCR achievement as a tool to individualise adjuvant systemic therapy, our results from multivariate analyses on the risk factors for BC recurrence unveiled the need of implementing new treatment strategies for patients at high risk of distant relapse. For patients with non-pCR, 14 cycles of T-DM1 have become a new adjuvant recommendation based on the results of the KATHERINE trial [26]. Extrapolation from data of the APHINITY trial hinted that continuing pertuzumab in the adjuvant setting is beneficial for patients with node-positive disease [27]. Otherwise, the phase III ExteNet trial showed benefits of 1-year additional anti-HER2 therapy with neratinib in patients with HR-positive disease previously treated with neoadjuvant trastuzumab-based therapy [28]. In the present study, no patient treated with adjuvant neratinib experienced distant recurrence, but it was administered in less than 2% of patients. Thus, neratinib offers a treatment option in high-risk HR-positive patients. However, Marin A. and collaborators recently showed that acquired secondary mutations in *HER2* promote resistance to neratinib [29], bringing out a different strategy to be considered in HER2-mutant BC.

Our data also suggest that dual HER2-blockade with pertuzumab and trastuzumab plus chemotherapy, administered in the neoadjuvant setting, is well tolerated in patients with HER-positive early BC. Anti-HER2 treatment-related toxicities were minimal, and grade 3–4 toxicities were only reported in six patients. All of them experienced LVSD, an expected cardiotoxic effect of trastuzumab and pertuzumab previously documented [30].

Limitations of this study include the absence of a control group and missing information on some variables due to the observational nature of the study. However, most patients had available data for the majority of study parameters. Safety information was limited, and mild or moderate toxicities were not collected. We also lack data on the ECOG performance of analysed patients, and more detailed treatment approaches. Despite these limitations, to our knowledge, this is the most extensive study on the effectiveness and safety of dual HER2-blockade plus chemotherapy in a real-world setting.

In conclusion, this study expands the knowledge on the effectiveness and safety of neoadjuvant pertuzumab and trastuzumab plus CT in real-world patients with early-stage HER2-positive BC and complements clinical trial data. Our results demonstrate that this neoadjuvant combination achieves high pCR rates. The pCR benefit is higher in HR-negative tumours and expressing higher levels of Ki-67. The distant relapse rate was low, and pCR achievement was associated with decreased BC recurrence. In addition, pCR was related to long-term survival outcomes, which remarks the urgent need of identifying novel biomarkers of the pCR. Finally, this neoadjuvant strategy showed an acceptable toxicity profile, with no unexpected safety issues.

## Data Availability

All data generated during this study are available from the corresponding author upon reasonable request.
